# Salidroside Attenuates Cognitive Dysfunction in Senescence-Accelerated Mouse Prone 8 (SAMP8) Mice and Modulates Inflammation of the Gut-Brain Axis

**DOI:** 10.3389/fphar.2020.568423

**Published:** 2020-12-09

**Authors:** Zeping Xie, Hui Lu, Sixia Yang, Yi Zeng, Wei Li, Linlin Wang, Guanfeng Luo, Fang Fang, Ting Zeng, Weidong Cheng

**Affiliations:** ^1^ Traditional Chinese Pharmacological Laboratory, School of Traditional Chinese Medicine, Southern Medical University, Guangzhou, China; ^2^ School of Traditional Chinese Medicine, Southern Medical University, Guangzhou, China; ^3^ Institute of Chinese Materia Medica, Shanghai University of Traditional Chinese Medicine, Shanghai, China

**Keywords:** microbiota, inflammation, microglia, SAMP8, cognition, salidroside

## Abstract

**Background:** Alzheimer’s disease (AD) is a fatal neurodegenerative disease characterized by progressive cognitive decline and memory loss. However, several therapeutic approaches have shown unsatisfactory outcomes in the clinical setting. Thus, developing alternative therapies for the prevention and treatment of AD is critical. Salidroside (SAL) is critical, an herb-derived phenylpropanoid glycoside compound, has been shown to attenuate lipopolysaccharide (LPS)-induced cognitive impairment. However, the mechanism underlying its neuroprotective effects remains unclear. Here, we show that SAL has a therapeutic effect in the senescence-accelerated mouse prone 8 (SAMP8) strain, a reliable and stable mouse model of AD.

**Methods:** SAMP8 mice were treated with SAL, donepezil (DNP) or saline, and cognitive behavioral impairments were assessed using the Morris water maze (MWM), Y maze, and open field test (OFT). Fecal samples were collected and analyzed by 16S rRNA sequencing on an Illumina MiSeq system. Brain samples were analyzed to detect beta-amyloid (Aβ) 1–42 (Aβ1-42) deposition by immunohistochemistry (IHC) and western blotting. The activation of microglia and neuroinflammatory cytokines was detected by immunofluorescence (IF), western blotting and qPCR. Serum was analyzed by a Mouse High Sensitivity T Cell Magnetic Bead Panel on a Luminex-MAGPIX multiplex immunoassay system.

**Results:** Our results suggest that SAL effectively alleviated hippocampus-dependent memory impairment in the SAMP8 mice. SAL significantly 1) reduced toxic Aβ1-42 deposition; 2) reduced microglial activation and attenuated the levels of the proinflammatory factors IL-1β, IL-6, and TNF-α in the brain; 3) improved the gut barrier integrity and modified the gut microbiota (reversed the ratio of Bacteroidetes to Firmicutes and eliminated Clostridiales and Streptococcaceae, which may be associated with cognitive deficits); and 4) decreased the levels of proinflammatory cytokines, particularly IL-1α, IL-6, IL-17A and IL-12, in the peripheral circulation, as determined by a multiplex immunoassay.

**Conclusion:** In summary, SAL reversed AD-related changes in SAMP8 mice, potentially by regulating the microbiota-gut-brain axis and modulating inflammation in both the peripheral circulation and central nervous system. Our results strongly suggest that SAL has a preventive effect on cognition-related changes in SAMP8 mice and highlight its value as a potential agent for drug development.

## Introduction

A prevalence-based study reported that the worldwide cost of dementia in 2015 was an enormous sum of US $818 bn, an increase of 35.4% compared to that in 2010 that was related to an increasing number of patients and rising costs per person (Wimo et al., 2017). The latest data from the United States show that the total costs for health care and hospice care for elderly people (≥65 years) with dementia in 2020 are estimated to be up to $305 billion (Alzheimer's disease facts and figures. Alzheimer's & dementia, 2020). Alzheimer’s disease (AD), as the primary cause of dementia, is a fatal neurodegenerative disease characterized by progressive cognitive decline and memory loss. The amyloid cascade hypothesis has been considered the main pathogenic concept in AD research for the past few decades. This hypothesis suggests that the accumulation of amyloid-*β* (A*β*) peptide in brain tissue is the primary event in AD, followed by the formation of neurofibrillary tangles (NFTs) containing tau protein (Hardy and Selkoe, 2002).

A number of recent studies have revealed the significance of the gut microbiota in AD. The microbiota-gut-brain axis has emerged as a potential key player that can have marked effects on AD pathology (Cryan et al., 2019). It has been confirmed that germ-free mice exhibit deficits in nonspatial and working memory, indicating that a commensal microbiota is required for cognition (Thion et al., 2018). It is widely accepted that the microbiota has a direct impact on the immune system, which is one of the various routes through which the microbiota communicates with the central nervous system (CNS). In addition, the microbiota has a profound impact on the maturation of microglial cells and effectively promotes the steady-state condition of microglia via secretion of short-chain fatty acids (SCFAs), highlighting the importance of the microbiota-gut-brain axis (Erny et al., 2015; Thion et al., 2018). On the other hand, there has been a growing number of recent studies investigating immune system-related events in AD, which have been shown to have strong pathogenic and therapeutic relevance (Heppner et al., 2015). Indeed, the inflammatory reaction that occurs in AD is driven mainly by CNS-resident immune cells, particularly microglia, which exert a dual influence in AD. It has been reported that disruption of the defense function of microglia leads to injury and even neuronal death (Hickman et al., 2018). Recent evidence strongly suggest that both the innate and adaptive immune systems are involved in AD, and it has been shown that proinflammatory mediators, including cytokines and chemokines, are increased in the peripheral circulation in individuals with AD (Cao and Zheng, 2019).

Animal models are paramount in AD research, especially for linking pathological changes, such as A*β* and tau accumulation. The senescence-accelerated mouse prone 8 (SAMP8) mouse strain, a spontaneous model of dementia, exhibits deficits in learning and memory abilities as well as pathological alterations in the brain, including increased oxidative stress, inflammation, A*β* accumulation and tau hyperphosphorylation (Currais et al., 2015). In contrast, its control, the senescence-accelerated mouse resistant 1 (SAMR1) line, ages normally. Salidroside (SAL), an herb-derived phenylpropanoid glycoside compound, has been shown to attenuate cognitive impairment in both lipopolysaccharide (LPS)-induced and d-gal-induced cognitive deficit models (Gao et al., 2015; Xu et al., 2019). Recent evidence has revealed that SAL provides neuroprotection by modulating mitochondrial biogenesis and microglial polarization (Barhwal et al., 2015; Liu et al., 2018). Notably, SAL exhibits significant anti-inflammatory effects in multiple diseases, such as osteoarthritis (Chen et al., 2019), colitis (Liu et al., 2019), skeletal muscle atrophy (Huang et al., 2019), renal interstitial fibrosis, (Li et al., 2019) and CNS diseases (Liu et al., 2018; Wang et al., 2018). Furthermore, recent findings have suggested that SAL alleviates liver injury by maintaining the balance of the gut microbiota (Yuan et al., 2019). Thus, it is reasonable to speculate that SAL exerts its neuroprotective function by regulating the gut microbiota, systemic inflammation, and subsequently neuropathologic changes. This study reports that SAL ameliorates cognitive decline in a reliable AD model, the SAMP8 mouse strain, and has beneficial effects on the inflammation-related microbiota-gut-brain axis.

## Materials and Methods

### Animals

A total of 10 male SAMR1 mice and 30 male SAMP8 mice weighing 27–32 g were used. The mice were obtained from the First Affiliated Hospital of Tianjin Traditional Chinese Medicine University (Tianjin, China). They were housed in a specific pathogen-free (SPF) level laboratory at Southern Medical University (Guangzhou, China) under standard conditions (22–23°C, 12-h light/dark cycle, and 60 ± 10% humidity) and provided with water and food ad libitum. They were adapted to their environmental conditions for 7 days before the experiments. All experimental protocols and animal handling procedures were conducted in strict accordance with the Guide for the Care and Use of Laboratory Animals published by the National Institutes of Health (NIH Publications No. 8023, revised in 1978). This study was approved by the Ethical Committee on Animal Experimentation of Southern Medical University.

### Experimental Designs

**FIGURE 1:**
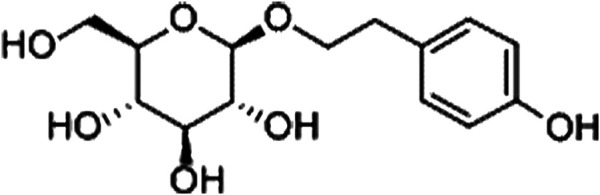
Molecular structure of Salidroside.

After being acclimatized to the laboratory conditions for 1 week, the 10 male SAMR1 mice were assigned to the control group (R1-Ct, treated with saline), and the 30 SAMP8 mice were randomly divided into the following three groups (10 mice/group): the model group (P8-Ct , treated with saline), the SALgroup (P8-SAL, treated with 50 mg kg^−1^ d^−1^ SAL) and donepezil (DNP) group (P8-DNP , treated with 1 mg kg^−1^ d^−1^ DNP). SAL (C_14_H_20_O_7,_ purity >98%) was obtained from Macklin Biochemical Co., Ltd. (Shanghai, China). DNP was supplied by Eisai Pharmaceutical Co., Ltd. (Tokyo, Japan). All mice were treated by daily gavage for 3 months, and tissues were then removed for other experiments. Every effort to minimize suffering was made Figure 1.

### Y-Maze

Spontaneous spatial working memory was assessed with a Y maze apparatus, which consisted of three identical arms (30 cm long, 8 cm wide, and 15 cm high). Each mouse was allowed to freely explore the three arms from the center of the maze for 5 min. The sequence of arm entries was recorded by a camera above the apparatus. A spontaneous alternation was defined as arm choices differing from the previous two choices (e.g., ABC, BCA, CAB, etc.). The alternation percentage (%) was calculated as the proportion of total spontaneous alternations relative to possible alternations (total arm entries −2) × 100%.

### Morris Water Maze (MWM)

To assess the hippocampus-dependent learning and memory abilities of the mice, the MWM test was performed after the Y maze. The MWM apparatus consisted of a blue circular pool with a diameter of 120 cm filled *2/3* with water (25°C ± 1) containing nontoxic ink and a circular platform (14 cm in diameter) submerged 1.5 cm below the water surface. The MWM test was performed as described previously (O'Neal-Moffitt et al., 2015). Briefly, during the acquisition trial (days 1–5), each mouse was trained to find the hidden platform within 60 s. The search trajectories were recorded with a camera, and the escape latencies were measured using DigBehv-Morris software (Shanghai, China). The time each mouse needed to find the platform within 60 s was defined as the escape latency. If a mouse did not find the platform successfully, the latency was recorded as 60 s, and the mouse was gently guided to the platform and allowed to stay on it for 10 s. A probe trial, in which the platform was removed, was performed on day 6. The number of target platform crossings and the time spent in each quadrant were recorded.

### Histological Analysis

Mice were anesthetized and intracardially perfused with cold PBS. Brain and intestinal tissues were carefully dissected and immersed in 4% paraformaldehyde at 4°C overnight. After being embedded in paraffin, the tissues were cut into sections (3 µm). Intestinal tissues were subjected to hematoxylin-eosin (HE) staining. Immunohistochemistry (IHC) analysis with a beta-amyloid 1–42 (A*β*_1-42_) antibody (ab201060-10, 1:1,000; Abcam) was performed on the brain sections following the standard IHC-paraffin protocol from Abcam. Pictures were taken with an MVX10 microscope (Olympus). For immunofluorescence (IF) analysis, brain sections were blocked with 5% BSA for 1 h and incubated with a primary antibody against CD68 (1:500; Servicebio) overnight at 4°C followed by an anti-rabbit Cy3-labeled secondary antibody for 1 h at room temperature. The nuclei were stained with DAPI reagent (Servicebio). For IF imaging, confocal microscopy (Zeiss, LSM800) was used, and images were taken and processed with ZEN software and Microsoft PowerPoint.

### Flow Cytometry

Flow cytometry was performed to detect the proportion of CD4^+^ or CD8^+^ lymphocytes in the spleen. Following sacrifice, the spleens were removed under sterile conditions, weighed, and processed into single cell suspensions, which then stained simultaneously with the following antibodies: PerCP-Cy^™^5.5 Rat Anti-Mouse CD3 (BD Biosciences, 560527), BV421 Rat Anti-Mouse CD4 (BD Biosciences, 562891), and FITC Rat Anti-Mouse CD8a (BD Biosciences, 553030), protected from light for 30 min. Labeled cells were fixed with 1% PFA and analyzed with a LSRFortessa X-20 flow cytometer (BD Biosciences, MA, United States) on FACSDiva 8.0.1 software (BD Biosciences). For each sample, corresponding isotype control antibodies were used. CD3^+^ cells were gated as T lymphocytes, and then the CD4^+^ and CD8^+^ populations were analyzed.

### Western Blot

Protein was extracted using the Whole Cell Lysis Assay (KeyGEN, Nanjing, China) according to the manufacturer's instructions. The proteins were separated using SDS‐PAGE and transferred to PVDF membranes (Millipore, Bedford, MA, United States). The membranes were blocked in 5% BSA for 1.5 h and then incubated with the following primary antibodies: anti-A*β*_1–42_ (ab201060-10, 1:1,000; Abcam), occludin (DF7504, 1:1,000; Affinity), ZO-1 (AF5145; 1:1,000; Affinity), *β*-actin (4,970; 1:1,000; CST), and GAPDH-HRP (HRP-60004, 1:8,000; Proteintech). After being washed with TBS‐T, the membranes were incubated with secondary antibodies (SA00001-2, 1:8,000; Proteintech) for 2 h at 4°C except when the GAPDH-HRP antibody was used. The protein signals were detected using an ECL system (Affinity, Jiangsu, China).

### Polymerase Chain Reaction

Total RNA was extracted from brain tissues using TRIzol reagent (Vazyme Biotech Co., Ltd) and converted to cDNA using the HiScript® III RT SuperMix for qPCR (+gDNA wiper) kit (Vazyme Biotech Co., Ltd.). qPCR was performed with ChamQ Universal SYBR qPCR Master Mix (Vazyme Biotech Co., Ltd.). All primers used are shown in Supplementary Table S1. Relative mRNA levels were calculated by normalization to the level of GAPDH (B661304, Sangon Biotech, Shanghai, China). Relative gene expression was analyzed based on the fold change (the 2 ^−ΔΔCt^ method) Table 1.

**TABLE 1:** Primers used for qPCR.

QPCR Timers	Sequence(5’-3’)
CD68-F	GAAATGTCACAGTTCACACCAG
CD68-R	GGATCTTGGACTAGTAGCAGTG
AIF1-F	ATTATGTCTTTGAAGCGAATGC
AIF1-R	TCTGAAGATGGCAGATCTCTTG
TNFα-F	ATGTCTCAGCCTCTTCTCATTC
TNFα-R	GCTTGTCACTCGAATTTTGAGA
IL6-F	CTCCCAACAGACCTGTCTATAC
IL6-R	CCATTGCAGAATGGAAAGTG
APP-F	TGAATGTGCAGAATGGAAAGTG
APP-R	AACTAGGCAACGGTAAGGAATC
IL-1β-F	TCGCAGCACATCAACAAGAG
IL-1β-F	AGGTCCACGGGAAAGACAGG
TREM2-F	TCATGTACTTATGAACGCCTGA
TREM2-R	GAGGTTCTTCAGAGTGATGGTG
TNF-F	ATGTCTCAAGCCTCTTCTCATTC
TNF-R	GCTTGTCAACTCGAATTTTGAGA

### 16S rRNA Sequencing and Data Analysis of Fecal Samples

Fecal samples were collected by using standardized collection procedures and were chosen at random. Five samples per group were used for 16S rRNA sequencing. The DNA samples were quantified, and then the V3-V4 hypervariable region was amplified using 338F (5′ACT​CCT​ACG​GGA​GGC​AGC​AG 3′) and 806R (5′GGACTACHVGGGTWTCTAAT 3′) primers. All PCR amplicons were concentrated and purified by gel electrophoresis and 3 µg of each amplicon was subsequently extracted. Sequencing was performed on the Illumina MiSeq system. The raw data were quality-filtered using QIIME (version 1.9.1). Operational taxonomic units (OTUs) were clustered by UPARSE (version 7.0.1090 http://www.drive5.com/uparse) with 97% similarity cut-off. The taxonomy of each 16S rRNA gene sequence was analysed using the RDP Classifier (https://sourceforge.net/projects/rdp-classifier) against the SILVA rRNA database (https://www.arb-silva.de) with 70% confidence threshold. PCoA were conducted according to the distance matrices. LEfSe analysis (linear discriminant analysis [LDA]) was conducted to calculate significant changes in relative abundance of microbial taxa between the groups.

### Multiplex Cytokine Assay

Serum was collected by centrifugation at 1000 *g* for 15 min at 4°C, aliquoted, and stored at −80°C until analysis. A Mouse High Sensitivity T Cell Magnetic Bead Panel (EMD Millipore, Billerica, MA, Unites States) was performed on the Luminex-MAGPIX multiplex immunoassay system according to the manufacturer’s instructions. The data were analyzed using Milliplex Analyst 5.1 software (EMD Millipore, Billerica, MA, United States).

### Statistical Analyses

Statistical analyses were performed with SPSS (IBM SPSS Statistics for Windows, version 20; IBM Corp., Armonk, NY, United States). Comparisons between groups were performed using one-way ANOVA for groups with one independent variables, and using two-way ANOVA for groups with two independent variables, followed by the least significant difference (LSD) test post hoc test. The following significance levels were used for comparisons between independent groups: #*p* < 0.05, ##*p* < 0.01, and ###*p* < 0.001 versus the R1-Ct group and *p < 0.05, **p < 0.01, and ***p < 0.001 versus the P8-Ct group. “ns” indicates no significant difference.

## Results

### Effect of SAL on the Behavioral Performance of SAMP8 Mice

**FIGURE 2:**
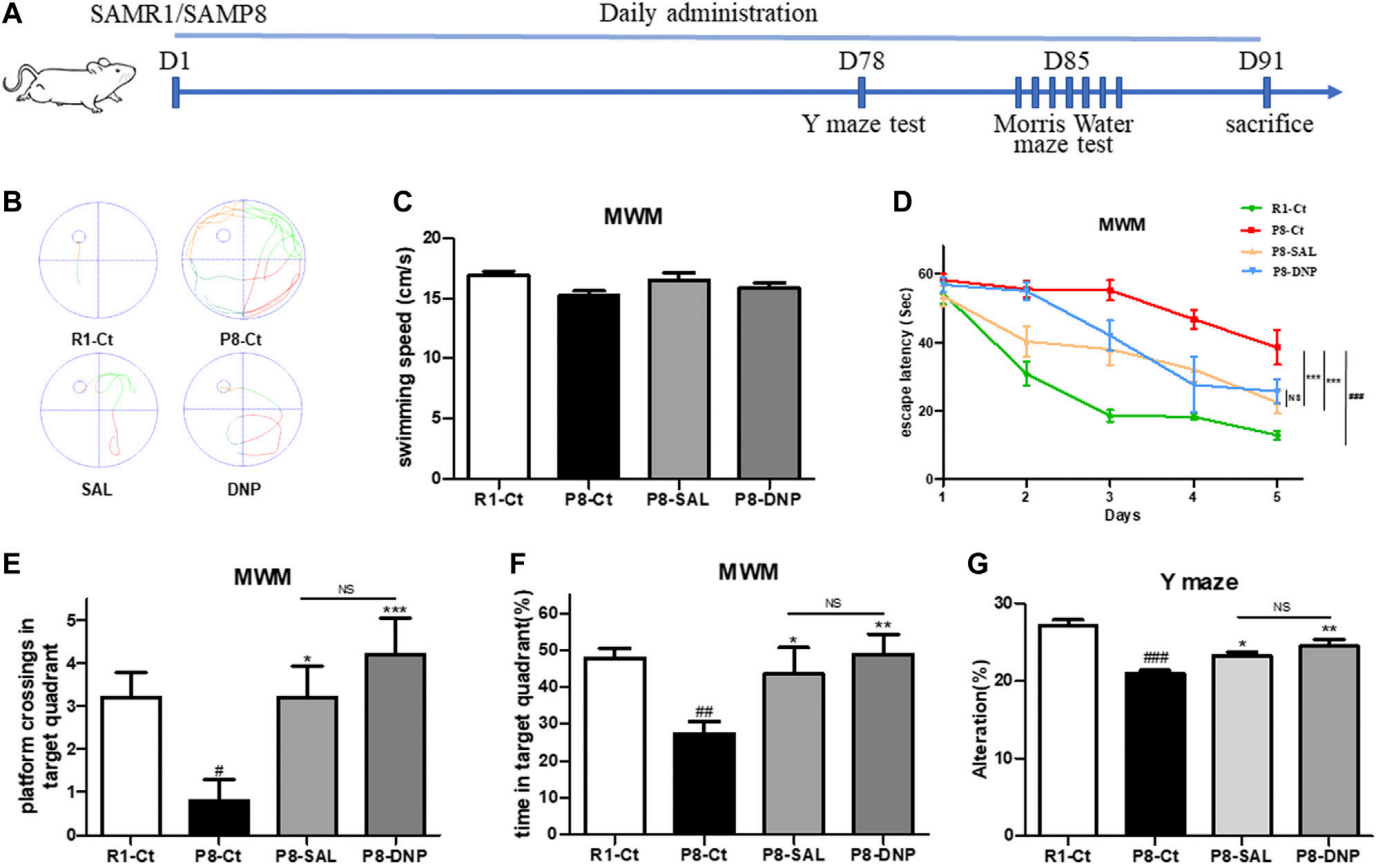
Effect of SAL on the behavioral performance of SAMP8 mice. **(A)** Schematic of the experimental design. **(B)** Representative automated traces from day 5 of the MMW are shown for each group. **(C)** The average swimming speed. **(D)** The escape latency to reach the hidden platform was measured during the 5-day training period. **(E,F)** In the probe trial, the number of platform crossings and the percentage of time that the mice spent in the target quadrant were analyzed (*n* = 5 for each group). **(G)** The percentage of correct alternations (alternation rate) in the Y maze test was calculated (*n* = 8 for each group). All data are shown as the mean ± SEM. One way ANOVA for **(D–F)**, two way ANOVA for **(C)**. #*p* < 0.05, ##*p* < 0.01, and ###*p* < 0.001 versus the R1-Ct group and *p < 0.05, **p < 0.01, and ***p < 0.001 versus the P8-Ct group. “ns” indicates no significant difference.

We treated mice with 50 mg kg^−1^ d^−1^ SAL, 1 mg kg^−1^ d^−1^ DNP or saline for 3 months, as described in the *Materials and Methods*. Treatment was started when the mice were 5 months of age, which is when AD pathological changes begin to emerge in this mouse strain (Pallas et al., 2008). To evaluate the effect of salidroside on the behavioral performance of SAMP8 mice, the Y maze test was conducted on day 78. Afterward, the MWM was performed for six consecutive days. Subsequently, the mice were sacrificed by euthanasia for histological and biochemical analyses (Figure 2A). The learning trials of the MWM revealed that SAMP8 required more time than SAMR1 mice to find the hidden platform and that escape latency was shortened by treatment with SAL or DNP (Figures 2B–D). Moreover, SAL treatment resulted in significant improvements in the percentage of time spent in the target quadrant and the number of target platform crossings, which represent memory recall, during the 60-s probe trial on the last day of the MWM (Figures 2E,F). Although the performances of P8-DNP mice were slightly better than those of P8-SAL mice, the difference was not significant (*p* > 0.05). In the Y maze test, the correct alternation rates of the SAL- and DNP-treated SAMP8 mice were significantly higher than the correct alternation rate of the model group (Figures 2G).

### Effect of SAL on Neurodegeneration and Neuroinflammation in SAMP8 Mice

**FIGURE 3:**
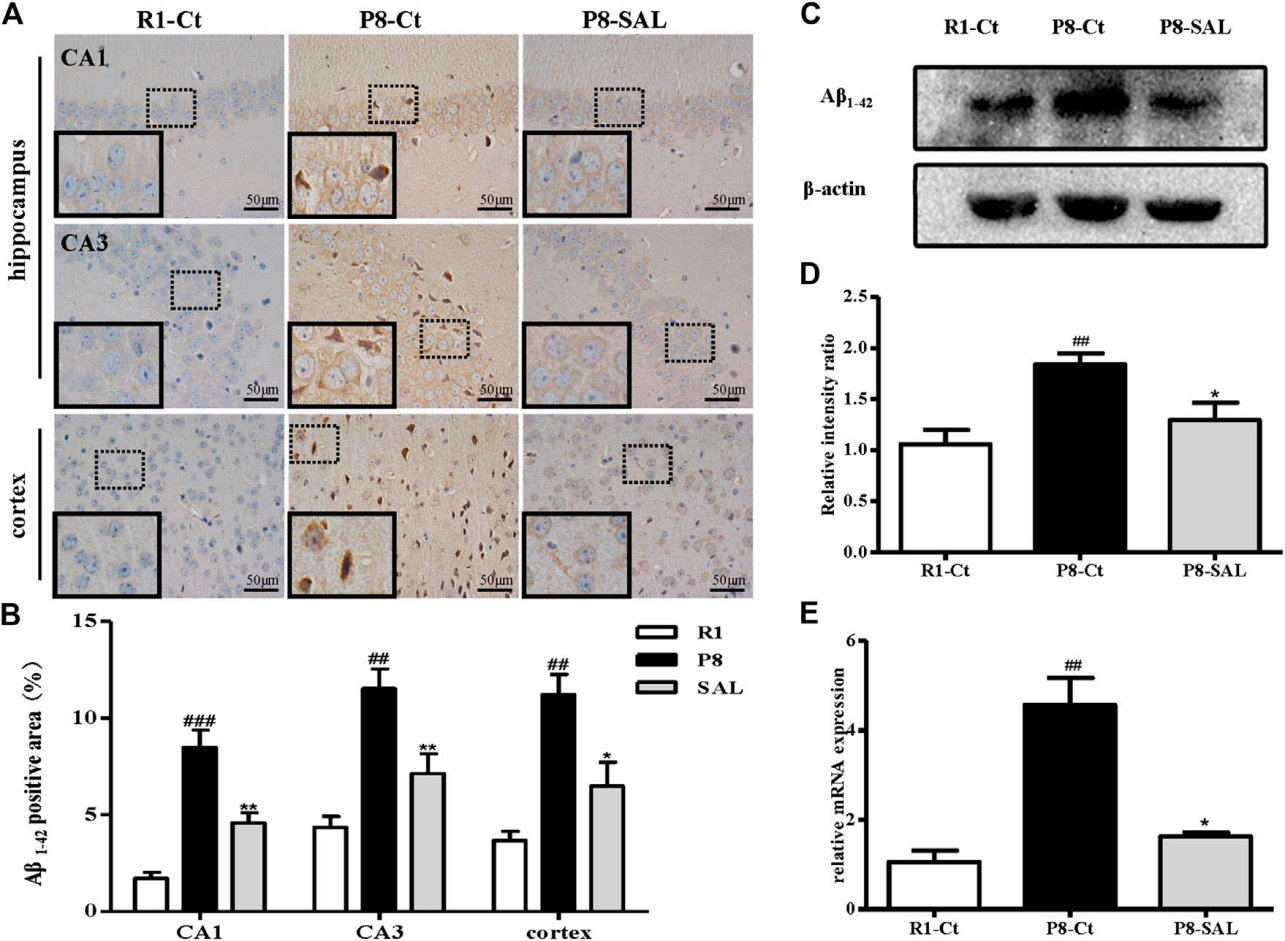
Effect of SAL on neurodegeneration in SAMP8 mice. **(A,B)** Immunohistochemical staining and quantification of Aβ1-42 in the hippocampus and cortex in each group (magnification ×400). The dotted box presents an enlarged image of the inset. **(C)** Western blot analysis of Aβ1-42 expression and quantification in the hippocampus. **(D)** mRNA levels of APP determined by qPCR. All data are shown as the mean ± SEM (*n* = 3 for each group). ^##^ *p* < 0.01, versus the R1-Ct group and *p < 0.05 versus the P8-Ct group.

The immunohistochemical results showed that A*β*_1-42_ was highly expressed in the hippocampi (CA1 and CA3 regions) and cortices of SAMP8 mice but showed almost no expression in SAMR1 mice. Due to their exposed hydrophobic surfaces, A*β*_1–42_ monomers and oligomers tend to interact with the neuronal membrane and cause toxicity (Narayan et al., 2013). However, SAL administration induced a reduction in neuronal damage resulting from A*β*_1–42_, which is thought to lead to an improvement in cognitive function, in SAMP8 mice (Figures 3A,B). The western blot analysis yielded results consistent with those of A*β*_1–42_ IHC (Figures 3C,D), and the mRNA expression of the amyloid precursor protein (APP) was also prevented by SAL (Figure 3E).

**FIGURE 4:**
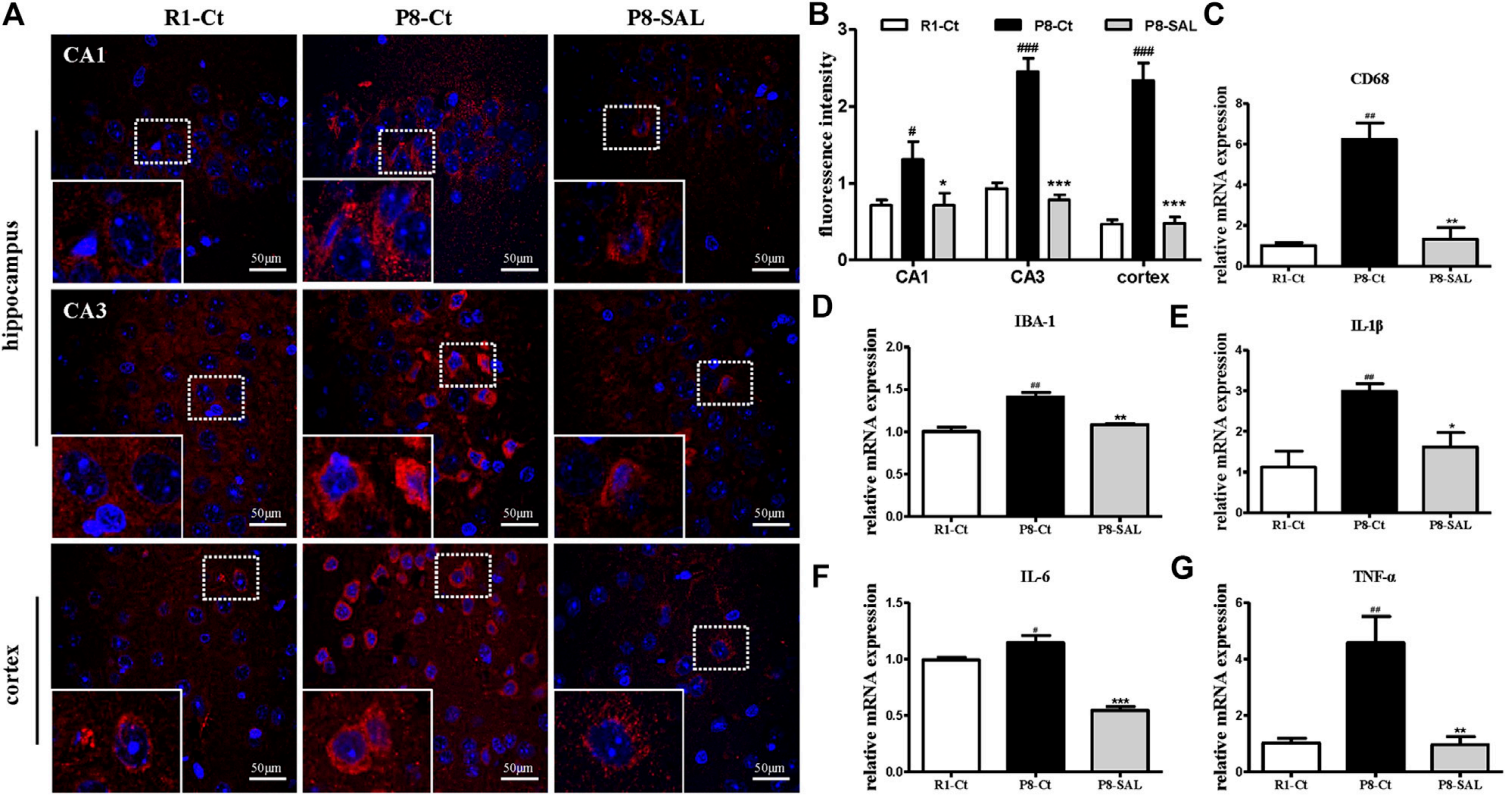
Effect of SAL on neuroinflammation in SAMP8 mice. **(A,B)** Representative images and quantification of immunofluorescence analysis using antibodies against CD68 (red) and DAPI staining (blue) in the hippocampus (CA1 and CA3 regions) and cortex (magnification ×400). The dotted box presents an enlarged image of the inset. The mRNA levels of **(C)** CD68, **(D)** IBA-1, **(E)** IL-1β, **(F)** IL-6 and **(G)** TNF-α,were determined by qPCR. All data are shown as the mean ± SEM (*n* = 3 for each group). ^#^ *p* < 0.05, and ^##^ *p* < 0.01 versus the R1-Ct group and *p < 0.05, **p < 0.01, and ***p < 0.001 versus the P8-Ct group.

We observed strong CD68 immunoreactivity in the P8-Ct group and fewer CD68-positive cells in the P8-SAL group than in the P8-Ct group, indicating that SAL reduced microglial activation in SAMP8 mice (Figures 4A, B). Consistently, the mRNA expression of CD68 and ionized calcium binding adaptor molecule 1 (IBA-1) was also prevented by SAL (Figures 4C,D). Moreover, the production of proinflammatory cytokines (interleukin (IL)-1β, IL-6, and TNF-α) was decreased in the SAL-treated group compared to the saline-treated group (Figures 4E–G). Our data revealed that SAL administration significantly reduced toxic A*β* peptide deposition in SAMP8 mice and that this effect was accompanied by a reduction in microglial neuroinflammation.

### Effect of SAL on the Intestinal Barrier and Gut Microbiota in SAMP8 Mice

**FIGURE 5:**
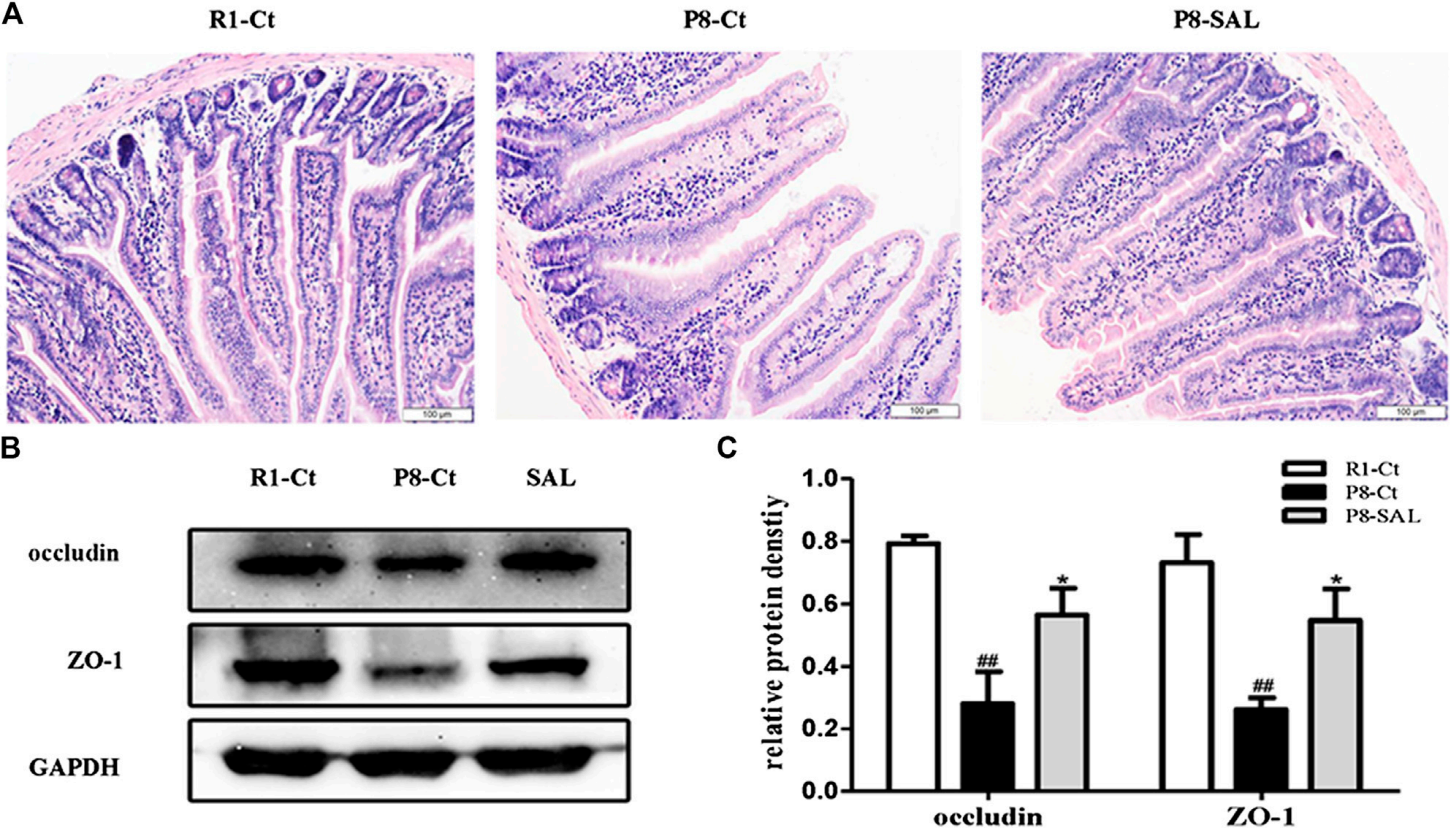
Effect of SAL on the intestinal barrier in SAMP8 mice. **(A)** HE staining of mouse intestinal tissues (scale bar = 100 μm). **(B)** Western blot analysis of ZO-1 and occludin in the intestine. **(C)** Protein quantification by western blotting. All data are shown as the mean ± SEM (*n* = 3 for each group). ^##^ *p* < 0.01 versus the R1-Ct group and *p < 0.05 versus the P8-Ct group.

Histological analysis and western blot analysis demonstrated that the integrity and tight junctions of the intestine were destroyed in SAMP8 mice. The HE staining of intestine showed that cells in the SAMP8 group were arranged irregularly, and edema was noted in villi (width of the villi). The intestinal mucosa of the P8-SAL group exhibited a more complete structure, less inflammatory infiltration in crypts, and less swelling of villi than the intestinal mucosa of the P8-Ct group (Figure 5 A), and the ZO-1 and occludin protein levels were higher in the P8-SAL group (Figures 5B,C).

**FIGURE 6:**
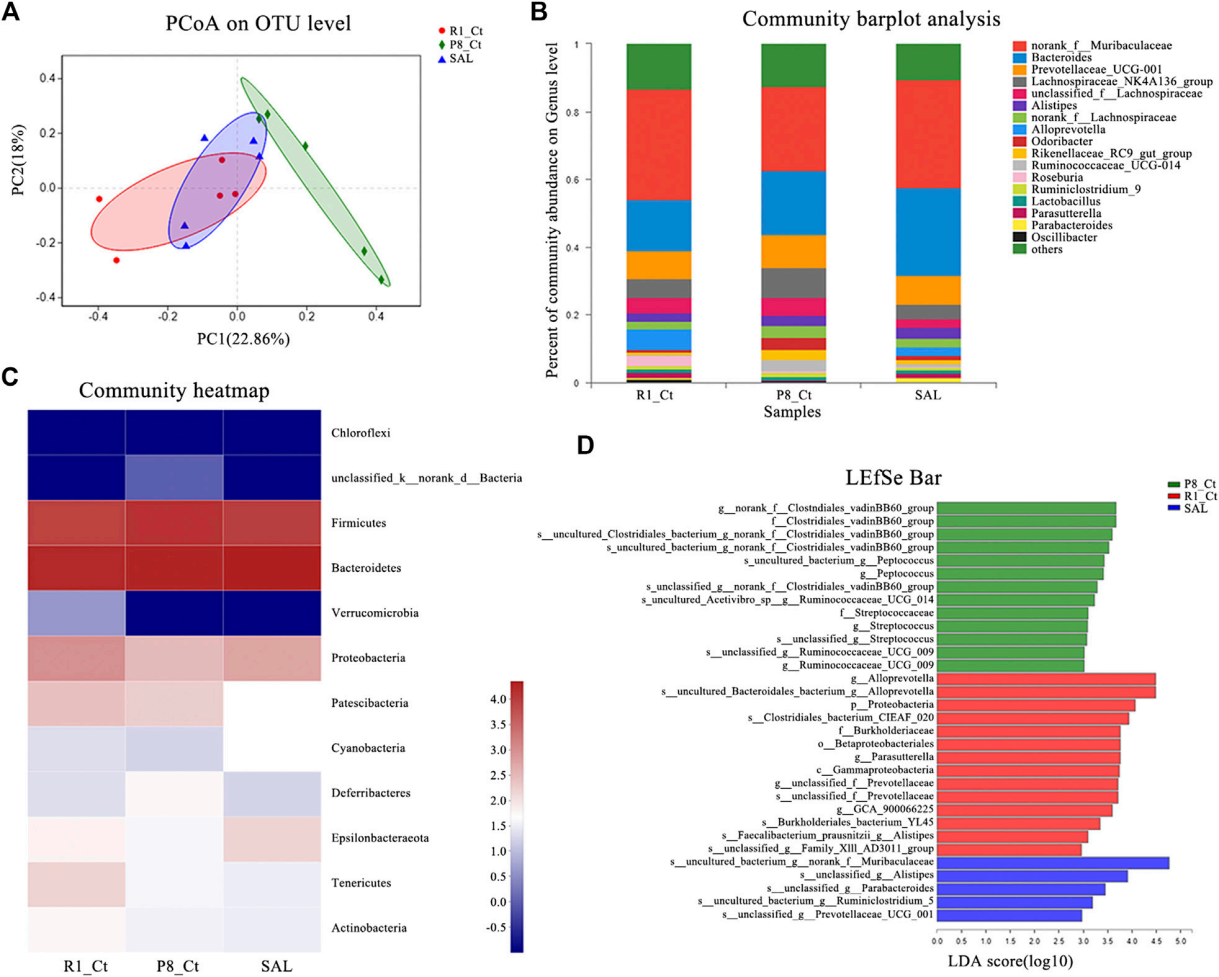
Effect of SAL on the gut microbiota in SAMP8 mice. **(A)** PCoA plots of the Bray-Curtis distance at the operational taxonomic unit (OTU) level. **(B)** Barplot analysis of the relative abundance in the community at the genus level. Genera with abundances less than 0.01 (1%) are summed in the category “others”. **(C)** Heatmap of microbial community abundance profiles at the phylum level. Top 50 species by total abundance. **(D)** Results of the LEfSe analysis (LDA > 2) at the phylum to the species level.

The sequence of the variable region of the 16S rRNA gene V3/V4 in fecal samples was analyzed. Fecal metagenomic sequencing data is available in the National Center for Biotechnology Information (NCBI) database with accession code PRJNA637826. To evaluate alterations in the microbial alpha diversity, we measured Chao, Shannon, Simpson, sob and ace diversity indices, which were not significantly different among groups (Supplemental Figure S1). Neither improvement in the total species diversity nor adverse effects was observed after SAL administration. Principal coordinate analysis (PCoA) revealed that the cluster from P8-SAL samples was more similar to that from R1-Ct samples, whereas the cluster from P8-Ct samples was more distinct (Figure 6A). To illustrate the differences in the microbiota composition, we conducted bar plot (Figure 6B), Circos (Supplemental Figure S2), and heatmap (Figure 6C) analyses. At the phylum level, there was a decrease in the *Bacteroidetes* phyla (R1-Ct, P8-Ct, and P8-SAL = 34, 30, and 36%, respectively) and an increase in the Firmicutes phyla (30, 40, and 29%, respectively), which are considered age-related differences that may also be associated with altered immune system function, in P8-Ct group (Nicholson et al., 2012). Interestingly, SAL treatment reversed the ratio of *Bacteroidetes* to *Firmicutes* to a level that was more similar to that observed in the R1-Ct group. Community barplot analysis at genus level showed that SAL administration increased the median abundance of *Norank_f_Muribaculaceae* (R1-Ct, P8-Ct, and P8-SAL = 37, 26, and 37%, respectively), *Alloprevotella* (70, 0.0, and 30%, respectively) and *Parasutterella* (48, 6.3, and 46%, respectively), and decreased the median abundance of *Prevotellaceae* (32, 37, and 31%, respectively), *Lachnospiraceae_NK4A136_group* (30, 46, 24%, respectively), *Unclassified_f_Lachnospiraceae* (35, 42, and 23%, respectively), *Alistipes* (28, 38, and 34%, respectively), *Norank_f_Lachnospiraceae* (26, 44, and 29%, respectively), *Odoribacter* (17, 55, and 28%, respectively), *Rikenellaceae_RC9_gut_group* (14, 61, and 25%, respectively), *Ruminococcaceae_UCG-014* (5.1, 78, and 17%, respectively) and *Ruminiclostridium_9* (28, 47, and 25%, respectively) in SAMP8 mice. A Circos diagram was used to visualize the associations between the abundance relationship between samples and bacterial communities at the genus level, which were consistent with the bar plot analysis results.

To further identify specific individual bacterial taxa that were differentially enriched among groups, we applied LEfSe analysis (Supplemental Figure S3). As shown above, significant enrichments in two families (*Clostridiales_vadinBB60* and *Streptococcaceae*), four genera (*norank_f_Clostridiales_vadinBB60*, *Peptococcus*, *Streptococcus*, and *Ruminococcaceae_UCG_009*) and seven species that were abolished by the SAL administration were identified in the SAMP8 mice, and five newborn species were present only in the P8-SAL group (Figure 6D). Venn diagram at the phylum, family, and genus levels. The analysis of the species in the Venn plots showed that salidroside basically eliminated the Chloroflexi phylum and five families and seven genera in SAMP8, and Corynebacterium was confirmed to be associated with AD-related pathological development (Supplemental Figure S4).

### Effect of SAL on Systematic Inflammation in SAMP8

**FIGURE 7:**
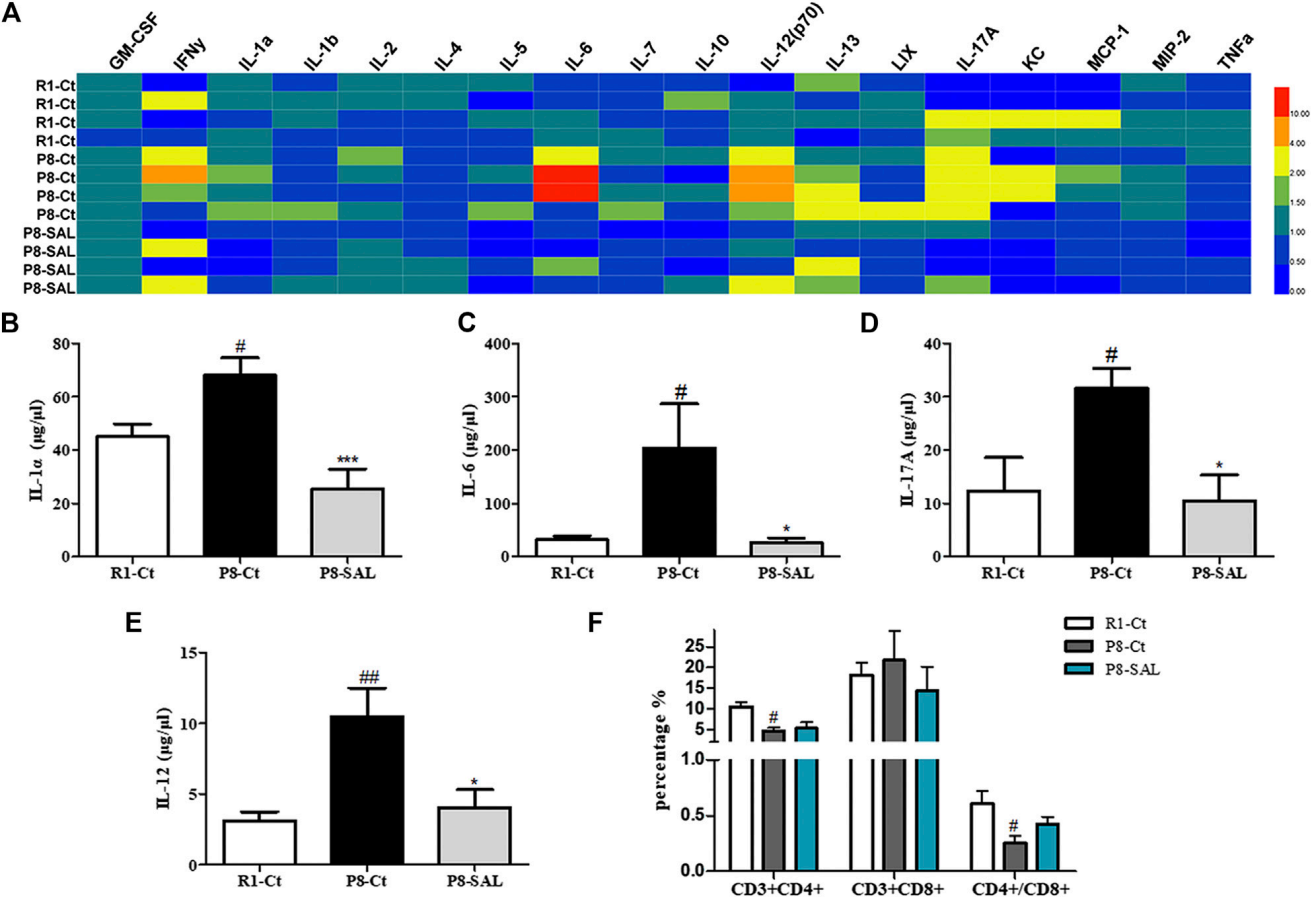
Effect of SAL on peripheral inflammation in SAMP8 mice. **(A)** Heatmap of 18 serum cytokine/chemokine levels, as detected by the Mouse High Sensitivity T Cell Magnetic Bead Panel. Quantitation of **(B)** IL-1α, **(C)** IL-6, **(D)** IL-12 and **(E)** IL-17A levels. **(F)** The spleen index was calculated as spleen weight **(G)**/mouse weight **(G)**. **(G)** Flow cytometry results, including the proportions of CD3^+^CD4^+^ and CD3^+^CD8^+^ cells and the ratio of CD4^+^/CD8^+^ spleen lymphocytes. The bars represent the mean ± SE of each group (*n* = 3). ^#^ *p* < 0.05, and ^##^ *p* < 0.01 versus the R1-Ct group and *p < 0.05 and ***p < 0.001 versus the P8-Ct group.

To assess the effects of SAL on peripheral cytokine secretion, a magnetic bead analysis approach was used to detect the concentration of 18 cytokines/chemokines in the plasma. The results showed that granulocyte-macrophage colony stimulating factor (GM-CSF), IL-1α, IL-6, IL-12, IL-13, and IL-17A were increased in SAMP8 mice compared to SAMR1 mice (Supplemental Figure S5) and that the levels of IL-1α (Figure 7B), IL-6 (Figure 7C), IL-17A (Figure 7D) and IL-12 (Figure 7E) were decreased after SAL administration compared to after saline administration. These data suggest that there is chronic inflammation in the circulation of SAMP8 mice and that SAL has significant anti-inflammatory properties in SAMP8 mice. In addition, the flow cytometry results demonstrated that the number of CD3^+^CD4^+^ lymphocytes and the CD4^+^/CD8^+^ ratio were significantly decreased in the spleens of SAMP8 mice compared to the spleens of SAMR1 mice. However, there were no significant changes in these measures after SAL administration compared to after saline administration (Figure 7F).

## Discussion

The senescence-accelerated prone mouse 8 (SAMP8) mouse strain is considered a reliable experimental model for studying the pathogenesis of and developing preventive and therapeutic strategies for age-related AD (Cheng et al., 2014). SAMP8 mice develop early deficits in learning and memory (at 5 months of age) accompanied by a number of AD-related brain alterations, including increased oxidative stress and tau phosphorylation (Pallas et al., 2008). Here, we treated 5-month-old SAMP8 with SAL for 3 months. At 8 months of age, the mice presented a stronger AD-related phenotype than SAMR1 mice, and the preventive effect of SAL was prominent. Our results suggest that SAL effectively alleviated hippocampus-dependent memory impairment in SAMP8 mice and did not have significantly different effects from those of DNP. Although DNP exerts a neuroprotective effect and is widely used in the treatment of AD, numerous studies have demonstrated that it causes adverse effects, including symptoms such as hostility, somnolence, fecal incontinence, nausea and rhinitis (Birks and Harvey, 2006; Lee et al., 2017; Adlimoghaddam et al., 2018). Previous studies have shown that SAL exerts neuroprotection, and no significant adverse effects have been reported yet (Zhang et al., 2010; Zhang et al., 2016; Zhong et al., 2018). Thus, SAL has the potential to be developed into an alternative treatment for AD.

**FIGURE 8:**
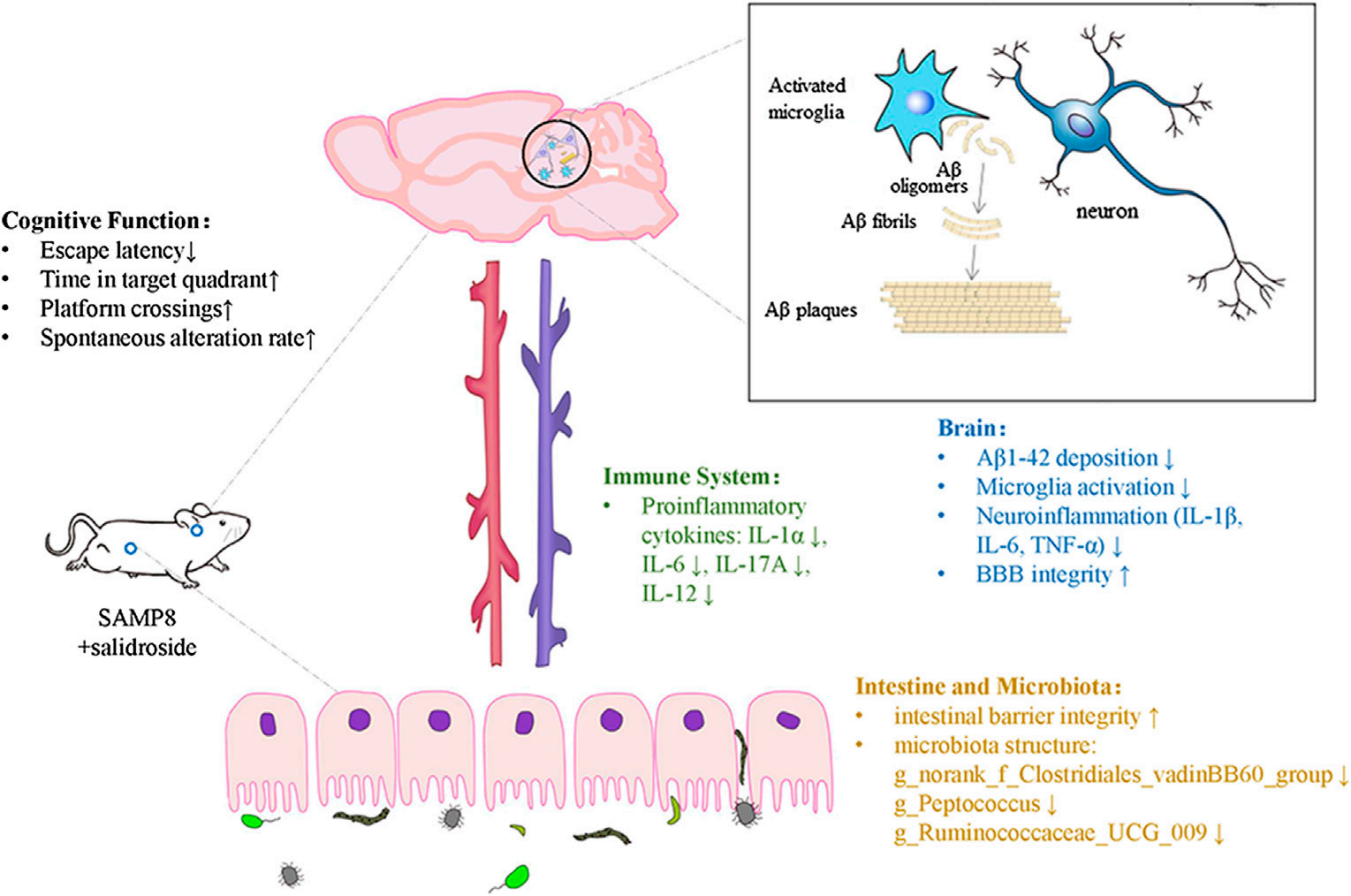
Salidroside modulates the Gut-brain Axis in SAMP8 mice.

In this study, SAL was shown to effectively attenuate both A*β*_1–42_ deposition and neuroinflammation in the brains of SAMP8 mice. Recent research in on a 3D noncell-autonomous cell culture model showed that a high Aβ42/40 ratio drives robust tau phosphorylation in human neurons, suggesting that selectively reducing the Aβ42/40 ratio could be a novel therapeutic approach (Kwak et al., 2020). In the present study, less A*β*_1–42_ deposition were observed in the hippocampi (CA1 and CA3 regions) and cortices of SAMP8 mice after SAL treatment compared to after saline treatment, confirming its therapeutic efficacy. Indeed, A*β*_1–42_ oligomers have a high tendency to attach to the membrane and have been implicated in neuronal injury and cognitive impairment associated with AD (Guglielmotto et al., 2014). Proinflammatory microglial activities are believed to have various detrimental effects on the brain and contribute to neurodegeneration. In particular, the activation of microglia increases the formation of Aβ oligomers and further aggregation (Narayan et al., 2013; Venegas et al., 2017). We showed here that the number of activated microglial cells, as determined by CD68 immunofluorescence, was significantly reduced in SAL-treated SAMP8 mice compared to saline-treated SAMP8 mice. Moreover, the mRNA levels of the proinflammatory cytokines IL-1β, IL-6, and TNF-α were decreased by varying degrees in SAMP8 mouse brains after SAL administration compared to after saline administration. These results therefore illustrate a novel effect of SAL involving attenuation of neuroinflammation in the AD brain Figure 8.

Multiple studies have indicated that microbial colonization of the gut is linked to dementia pathogenesis via detrimental effects on metabolic disorders or low-grade inflammatory progression, leading to brain damage (Alkasir et al., 2017). Consistent with previous studies (Peng et al., 2018), our findings provide further evidence that the microbiota-gut-brain axis may be involved in AD-like pathogenesis in SAMP8 mice. PCoA and microbiota composition analysis revealed that the cluster from salidroside-treated SAMP8 mice was more similar to that from SAMR1 mice, whereas the cluster from untreated SAMP8 mice was the most distinct. In our study, a shift in the ratio of *Bacteroidetes* to *Firmicutes*, which is one of the classic age-related changes in microbiota composition that is associated with increased inflammation, was observed in SAMP8 mice (Nicholson et al., 2012; Cowan et al., 2014). The reversal effect of SAL indicated its potential to delay senescence and reduce inflammation. Significant enrichments in two families (*Clostridiales_vadinBB60* and *Streptococcaceae*), four genera (*norank_f_Clostridiales_vadinBB60*, *Peptococcus*, *Streptococcus*, and *Ruminococcaceae_UCG_009*) and seven species, which were abolished by SAL administration, were identified in SAMP8 mice,. These results suggest that SAL normalizes alterations in the intestinal microbiota. It should be noted that *Clostridiales* and *Streptococcaceae* are part of the phylum *Firmicutes* and may be associated with cognition (Bajaj et al., 2019). In addition, five newborn species were present only in the P8-SAL group, but whether they are beneficial requires further study.

Given the importance of microglial functions in the promotion of neurodegenerative processes, it is reasonable to speculate that changes in the gut microbiota, which are capable of inducing inflammation via some cell components or metabolites, may influence these inflammatory and degenerative alterations in the AD brain. In our study, SAL was able to restore intestinal barrier integrity, which may result in less accumulation of microbial products in the periphery as well as a reduction in chronic inflammation. As immune-related effects on bacteria are important steps toward understanding bacterial contributions to cognition, we estimated alterations in the circulating levels of proinflammatory and anti-inflammatory cytokines/chemokines, which directly affect brain function. Low-grade systemic inflammation was observed in the serum of SAMP8 mice, whereas SAL decreased the levels of the proinflammatory cytokines IL-1α, IL-6, IL-17A and IL-12. Notably, elevated levels of these four proinflammatory cytokines are reported to be involved in cognitive decline or AD pathology (Veerhuis et al., 2003; Vom Berg et al., 2012; Fragoulis et al., 2017; Italiani et al., 2018). Th1 cells significantly accelerate markers of AD, as demonstrated primarily in murine models (Lambracht-Washington et al., 2011), and *in vivo* imaging experiments have shown that Th17 cells induce severe fluctuations in the neuronal intracellular Ca (2+) concentration, causing neuronal damage and neuroinflammation (Siffrin et al., 2010). It is known that IL-12 induces T lymphocytes to differentiate into Th1 cells and that IL-17A is a Th17-specific cytokine. Additionally, the pleiotropic cytokine IL-6 is crucial for the differentiation of Th17 cells. Indeed, there are associations between serum profiles of inflammatory factors and gut microbiomes. One study suggested that variability of the microbiota, especially the phylum *Proteobacteria*, is positively correlated with IL-6 (Biagi et al., 2010). Moreover, it has been reported that blocking IL-1α leads to a modification in the gut microbiome and effectively reduces inflammation and damage in a mouse model of Crohn’s-like ileitis (Menghini et al., 2019). Microbial products have direct effects on the immune system, which affects brain function through circulating cytokines (Cryan and Dinan, 2012). It has been reported that many of the beneficial effectual of bacteria on learning and memory occur alongside reductions in proinflammatory cytokines (Wang et al., 2015; Allen et al., 2016; Burokas et al., 2017). One study showed that systemic immune alterations trigger and drive the development of AD-related neuropathology, specifically Aβ accumulation and tau phosphorylation, as well as microglia and gliosis activation in wild-type mice, suggesting that immune reactions can precede AD-related pathology and may even be sufficient to cause it (Krstic et al., 2012). Thus, the effect of SAL on cognition may be associated with recovery of the gut microbiota composition, which is crucial for reducing peripheral low-grade inflammation thus improving of brain-blood barrier function and suppressing neuroinflammatory stimuli.

In summary, SAL reversed the AD-related changes in the SAMP8 mice potentially by regulating the microbiota-gut-brain axis and modulating inflammation in both the peripheral circulation and central nervous system. Our results strongly suggest that SAL has a therapeutic effect on cognition-related changes in SAMP8 mice and highlight its value as a potential agent for drug development.

## Data Availability Statement

The datasets presented in this study can be found in online repositories. The names of the repository/repositories and accession number(s) can be found below: https://www.ncbi.nlm.nih.gov/, PRJNA637826.

## Ethics Statement

The animal study was reviewed and approved by the Ethical Committee on Animal Experimentation of Southern Medical University.

## Author Contributions

ZX designed and performed the experiments, analyzed the data, and drafted the manuscript. HL collected the fecal samples and assisted with the 16S rRNA sequencing analysis. SY, YZ, FF, and TZ assisted with the histological staining and qPCR. LW designed the flow cytometry experiment and assisted with the flow cytometry data acquisition and analysis. LW and GL assisted with the behavioral tests and western blot analysis. FF assisted with the multiplex immunoassay experiments. WC provided study supervision and manuscript revision. All authors read and approved the final manuscript. All authors contributed to the article and approved the submitted version.

## Funding

This research was funded by the Guangzhou Municipal Science and Technology Project (grant number 201904010168), the National Natural Science Foundation of China (grant number 81973641), and the National Key Research and Development Program of China (grant number 2018YFC1704400).

## Conflict of Interest

The authors declare that the research was conducted in the absence of any commercial or financial relationships that could be construed as a potential conflict of interest.

## Abbreviations

**Abbreviations** AD Alzheimer’s disease, SAL salidroside, SAMP8 senescence-accelerated mouse prone eight, Aβ beta-amyloid, NFTs neurofibrillary tangles, CNS central nervous system, SCFAs short-chain fatty acids, SAMR1 senescence-accelerated mouse resistant 1, OFT open field test, MWM Morris water maze, HE hematoxylin-eosin, IHC immunohistochemistry, IF immunofluorescence, IBA-1 ionized calcium binding adaptor molecule 1, APP amyloid precursor protein, IL interleukin, PCoA principal coordinate analysis, Th helper T cell, GM-CSF granulocyte-macrophage colony stimulating factor.
