# Supplementary material for: Salidroside Attenuates Cognitive Dysfunction in Senescence-Accelerated Mouse Prone 8 (SAMP8) Mice and Modulates Inflammation of the Gut-Brain Axis
Source: Front Pharmacol. 2020 Dec 9;11:568423. doi: 10.3389/fphar.2020.568423 (PMC7759146; doi:10.3389/fphar.2020.568423)
Supplement: Supplementary file 1 [file datasheet1.docx]

Supplementary Material

# Supplementary Figures


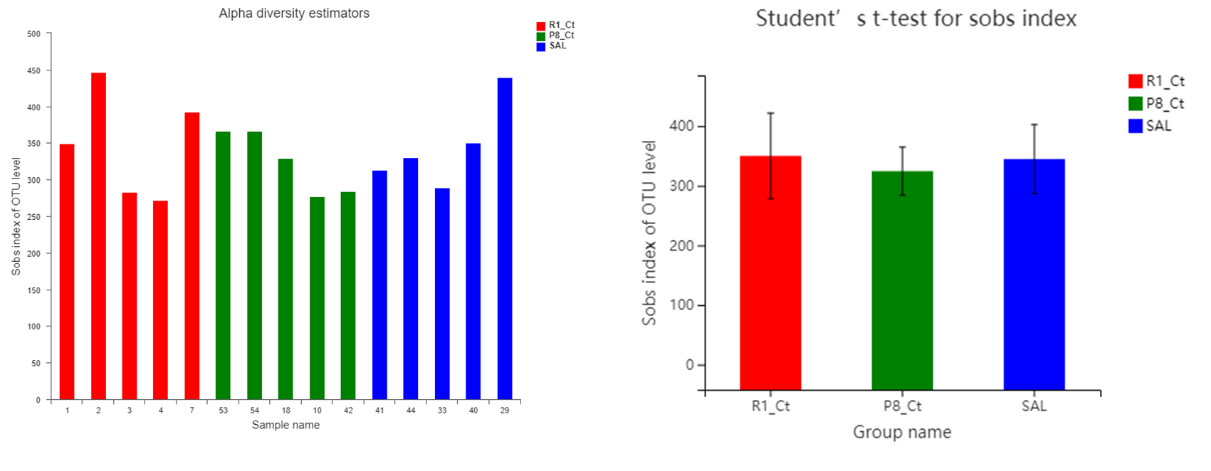


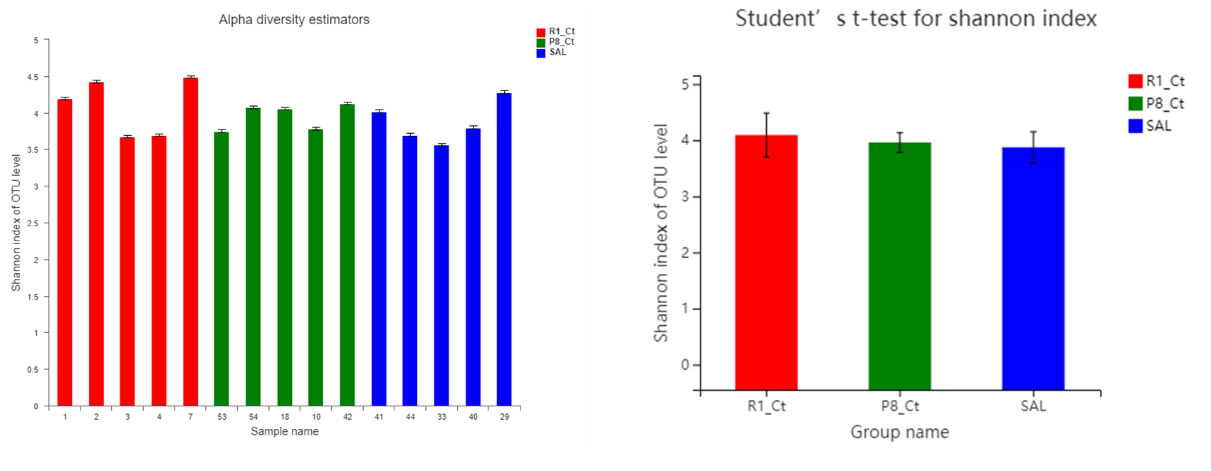


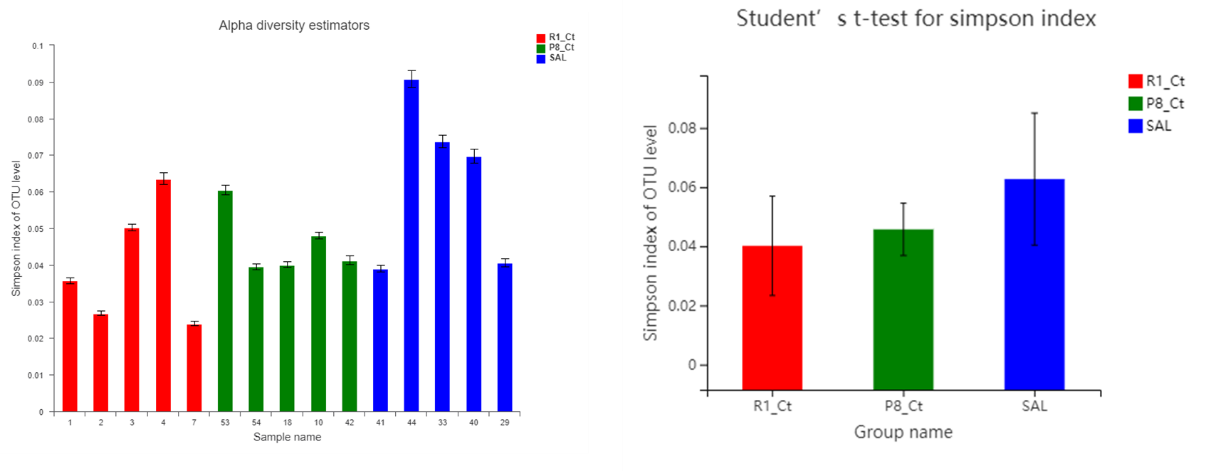


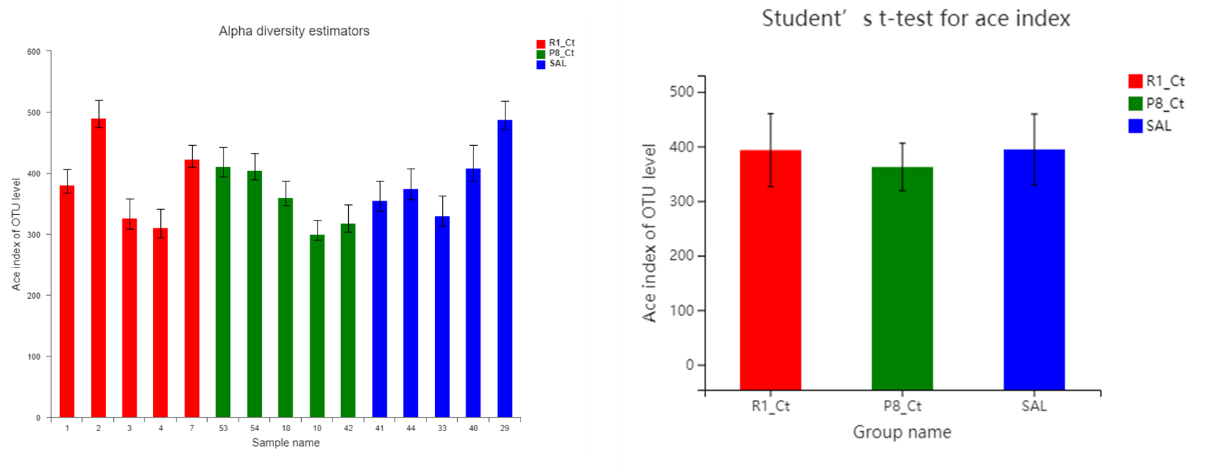


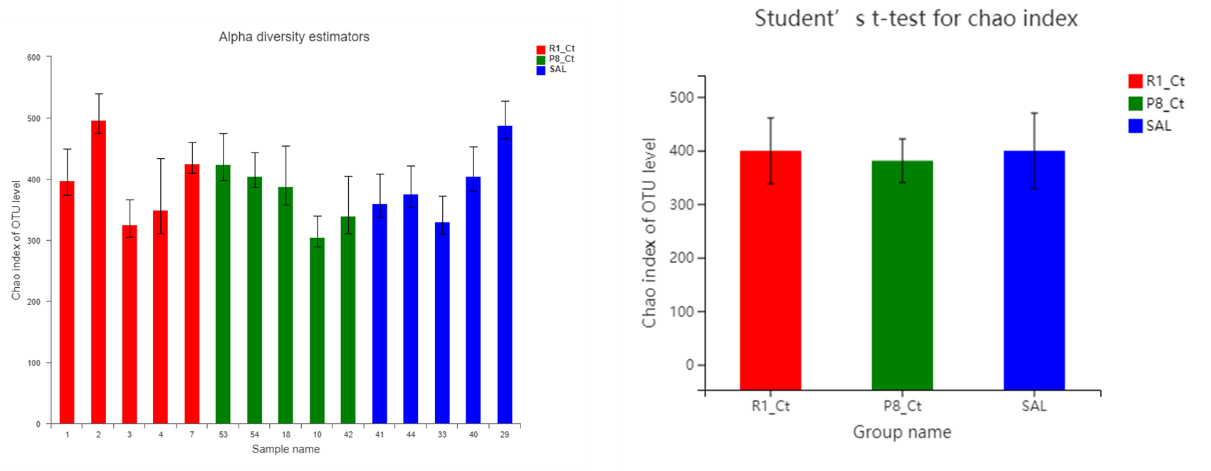


**Supplementary Figure 1.** To evaluate alterations in the microbial alpha diversity, we measured Chao, Shannon, Simpson, sob and ace diversity indices, which were not significantly different among groups.


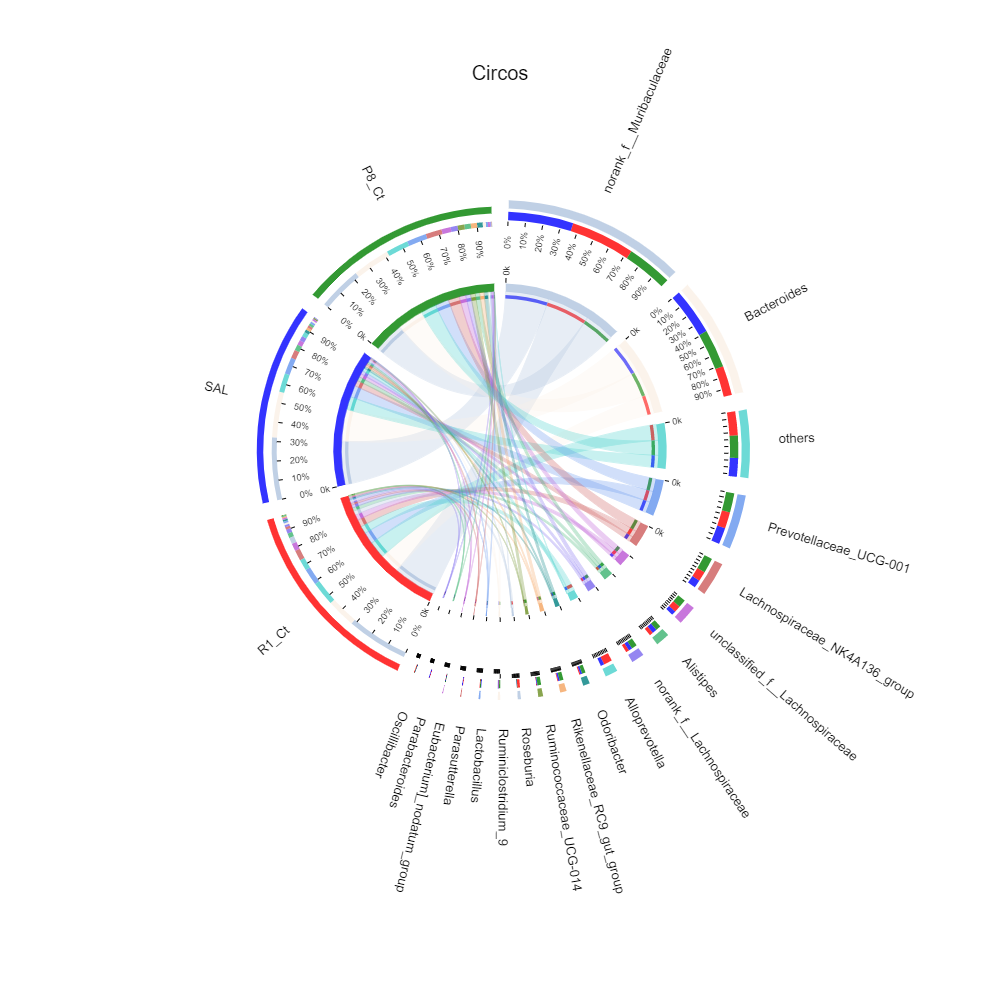


**Supplementary Figure 2.** A Circos diagram was used to visualize the associations between the abundance relationship between samples and bacterial communities at the genus level, which were consistent with the bar plot analysis results.


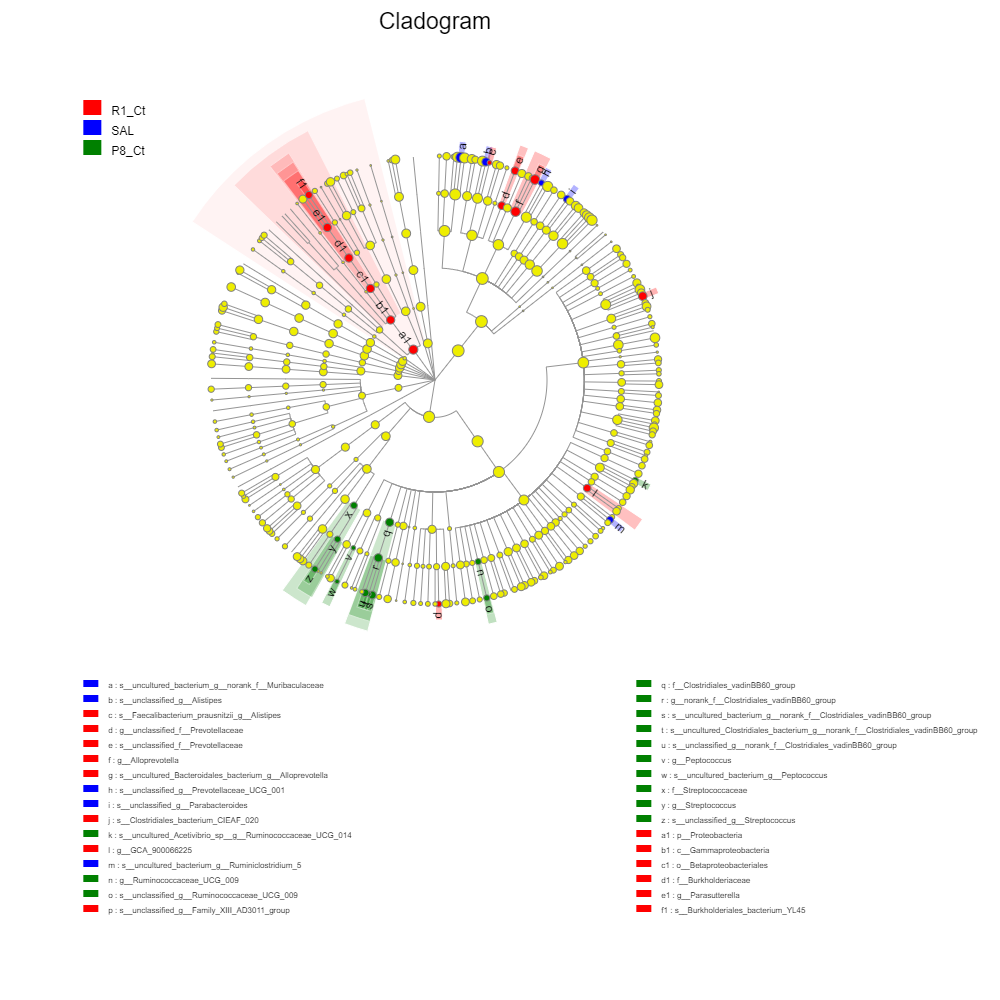


**Supplementary Figure 3.** To further identify specific individual bacterial taxa that were differentially enriched among groups, we applied LEfSe analysis


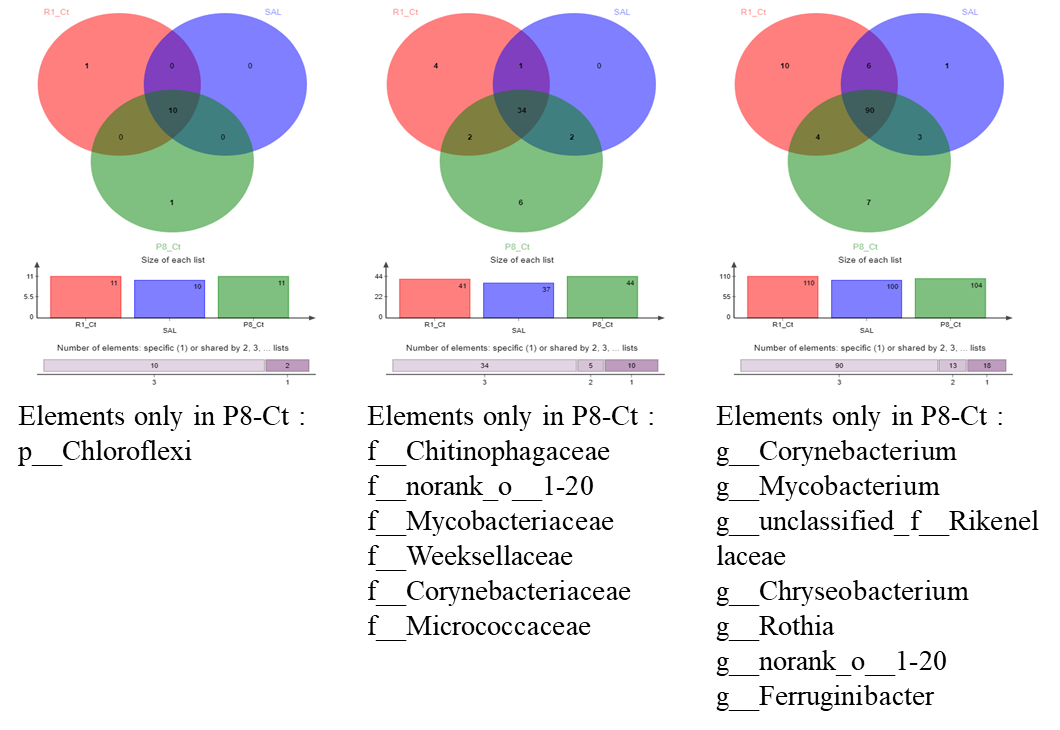


**Supplementary Figure 4.** Venn diagram on the phylum, family, and genus levels. Analysis of species venn plots showed that salidroside has basically eliminated the Chloroflexi phylum in SAMP8, as well as 5 families and 7 genera, of which Corynebacterium has been confirmed to be associated with AD-Related pathological development


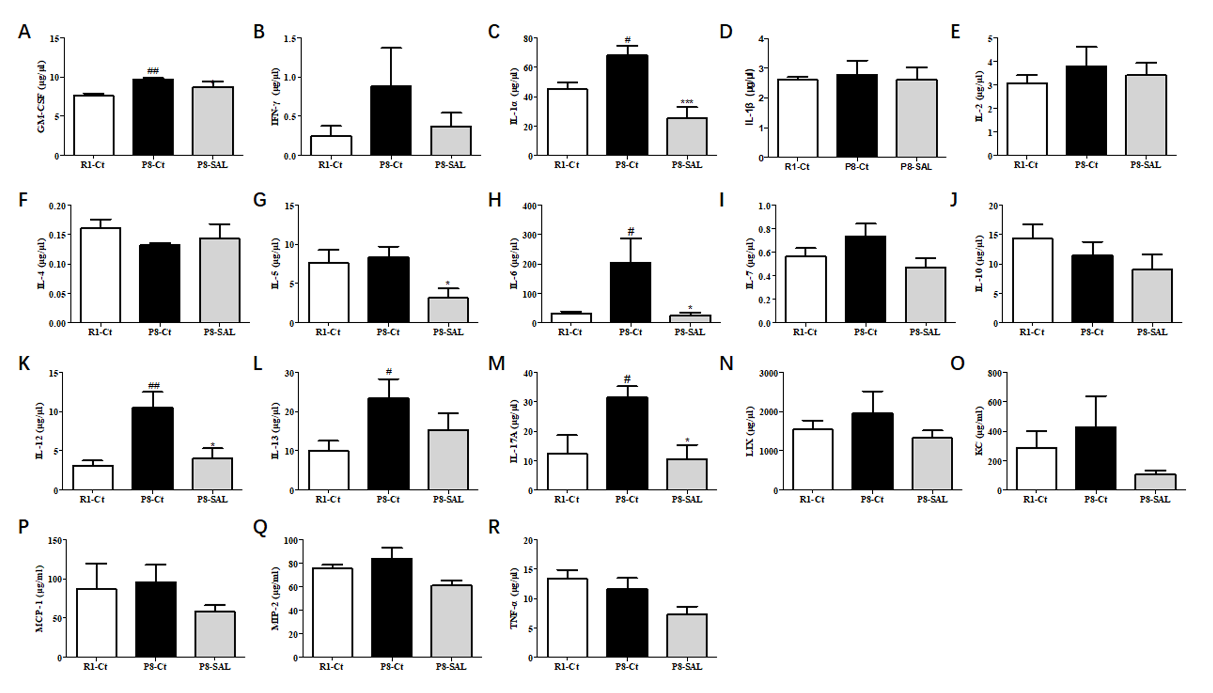


**Supplementary Figure 5.** To assess the effects of SAL on peripheral cytokine secretion, a magnetic bead analysis approach was used to detect the concentration of 18 cytokines/chemokines(GM- CSF,IFN-γ,IL-1α,IL-1β,IL-2,IL-4,IL-5,IL-6,IL-7,IL-10,IL-12,IL-13,LIX, IL-17A,KC,MCP-1,MIP-2,TNF-α) in the plasma.
